# Antibacterial and anti-inflammatory effects of PGLa-loaded TiO_2_ nanotube arrays

**DOI:** 10.3389/fchem.2023.1210425

**Published:** 2023-06-08

**Authors:** Bin Xuan, Lei Li, Hui Zhang, Zhuojue Liu, Ruxi Luo, Wenpeng Yang, Weili Wang

**Affiliations:** Department of Stomatology, Aerospace Center Hospital, Beijing, China

**Keywords:** TiO_2_ nanotube arrays, Ti substrates, PGLa-loaded, antibacterial, anti-inflammation

## Abstract

**Objectives:** This study investigated the antimicrobial effect and anti-inflammatory activities of PGLa-loaded TiO_2_ nanotube arrays (TiO_2_ NTs) in osteoblast-like MG-63 cells.

**Methods:** The surface morphology and roughness of three titanium (Ti) substrates (Ti, TiO_2_ NTs, PGLa-loaded TiO_2_ NTs) were evaluated by scanning electron microscopy (SEM) and atomic force microscope (AFM). The wettability of three titanium substrates was evaluated by contact angle. Biocompatibility of PGLa-loaded TiO_2_ NTs were evaluated in MG-63 cells (cell adhesion, proliferation, cytoskeletal evaluation and alkaline phosphatase activity). Spread plate counting method was used to evaluate antibacterial abilities of the titanium substrates. The calcein AM/PI staining evaluated cell viability of MG-63 cells on the substrates with or without proinflammatory factors (TNF-α).

**Results:** The average surface roughness of untreated Ti, TiO_2_ NTs, PGLa-loaded TiO_2_ NTs were found to be 135.8 ± 6.4 nm, 300.5 ± 10.5 nm, 348.9 ± 16.9 nm, respectively. The contact angle of the untreated Ti was 77.4° ± 6.6°. TiO_2_ NTs displayed excellent wettability which of contact angle was 12.1° ± 2.9°. The contact angle of the PGLa-loaded TiO_2_ NTs was 34.6° ± 4.9°. MG-63 cells on surface of PGLa-loaded TiO_2_ NTs showed better cell adhesion, proliferation and osteogenic activity. The antibacterial rate of PGLa-loaded TiO_2_ NTs group significantly increased (84.6% ± 5.5%, *p* < 0.05). The rate of dead cells on the surfaces of the PGLa-loaded TiO_2_ NTs with TNF-α decreased significantly (4.49% ± 0.02, *p* < 0.01).

**Conclusion:** PGLa-loaded TiO_2_ NTs have multi-biofunctions including biocompatibility, antibacterial and anti-inflammatory properties.

## 1 Introduction

The application of dental implants helps patients with missing teeth recover function and beauty. Although dental implant technology is becoming increasingly mature, complications may occur in both the long and short term (Alghamdi and Jansen, 2020). Three factors are critical to the success of implants: successful osseointegration (Staehlke et al., 2020), reduction of the degree of peri-implant inflammation, and reduction of bacterial adhesion on the implant surface (Del et al., 2020). Therefore, in order to prolong the service lives of implants, it is essential to develop new implant surface modification technology with enhanced osseointegration, antibacterial, and anti-inflammatory functions.

Because of its excellent performance in different aspects, Titanium (Ti) is very suitable for dental implant materials. However, the early failure of Ti implants can occur due to poor osseointegration. This issue can be addressed by modifying Ti surfaces that promote osseointegration. Previous studies had found that a porous implant surface structure can provide a clearer and more reliable rough surface to enhance bone integration (Roguska et al., 2012). The porous structure could be used as drug loading space, and the sustained-release effect could be achieved after appropriate improvement (Arruebo, 2012). TiO_2_ nanotube arrays (TiO_2_ NTs) as the porous Ti dioxide layer were formed electrochemically in the fluorinated electrolyte, which had chemical stability and good biocompatibility. TiO_2_ NTs structures increased the roughness on Ti surfaces; this characteristic had significant impacts on cell adhesion, migration, proliferation and differentiation (Minagar et al., 2013). Histological experiments showed that TiO_2_ NTs could increase the osteoblast adhesion of rabbits by nine times and increase the mineralization rate by approximately three times. With improved osseointegration, TiO_2_ NTs reduced the initial adhesion and colonization of the staphylococcal. TiO_2_ NTs showed great potential to enhance the surface properties of Ti implants (Su et al., 2018). They improved the cytological behaviors of human periodontal ligament stem cells (PDLSc), such as adhesion, morphology, proliferation, and osteogenic differentiation. Compared with Ti control, the coarser TiO_2_ NTs exhibited better hydrophilicity and protein adsorption ability. Therefore, the surface modification of TiO_2_ NTs can improve the activity of osseointegration (Li et al., 2017).

The pathogenesis of peri-implantitis was that microorganisms around an implant caused continuous damage to the surrounding alveolar bone. Oral microorganisms adhered to various types of surfaces by producing a variety of adhesion factors. The inherent characteristics of materials affected the number of microorganisms and the adhesion of material surface. An ideal dental implant surface should enhance osseointegration, it had anti-inflammatory and antibacterial functions to facilitate hard and soft tissue stability. Osteoblast played an important role in the renewal of bone tissue around the implant and affected the long-term stability of the implant. TiO_2_ NTs as porous layers have become promising for use in implantable drug delivery systems. The NT structure on the implant surface served as a storage space for drugs, directly transported drugs to the alveolar bone around the implant. It was previously found that the osseointegration on the implant surface can be improved by loading recombinant human bone morphogenetic protein-2 (rhBMP-2) into TiO_2_ NTs (Lee et al., 2015). The human osteoblast like MG-63 cell line was commonly used for bone research. Previous scholars had successfully utilized this cell to establish models for studying cell material interactions, including cell adhesion, proliferation and signal transduction on biomaterials (Lai et al., 2015). PGLa as the cationic amphiphilic peptide showed biological regulation and antibacterial activity (Zasloff, 1987). This peptide was harvested from the granular glands of the South African clawed frog (Giovannini et al., 1987). In this research, the antibacterial and inflammatory properties of PGLa-loaded TiO_2_ NTs were investigated for the first time.

In the present study, TiO_2_ NTs were formed on a Ti substrate by anodic oxidation. PGLa was loaded on the surface of TiO_2_ NTs. The main purpose of this study was to explore the antibacterial and anti-inflammatory effects of PGLa-loaded TiO_2_ NTs. The surface topography and wettability were evaluated, and biocompatibility such as cell adhesion, proliferation and osteogenic activity were also studied.

## 2 Materials and methods

### 2.1 Anodizing and loading of PGLa into TiO_2_ NTs

The chemicals used in this experiment were used without further purification and were of the highest available purity. We fabricated TiO_2_ NTs on the pure Ti foils (10 mm diameter round). The Ti foils were washed in an ultrasonic bath mixed with acetone, isopropanol, and ethanol; washed with deionized water; and dried. Electrochemical anodization was performed in a double electrode cell at room temperature. The distance between the two electrodes was 4 cm. A Ti sheet and graphite plate were used as the anode and cathode, respectively. We dissolved 0.27 M NaF in ethylene glycol to prepare the electrolytes. The Ti foils were continuously applied at 40 V for 1 h. The as-prepared TiO_2_ NTs were washed with ethanol and distilled water and dried at 450°C for 2 h. The PGLa drugs (20 μg/ml, MedChemExpress, United States) were loaded into TiO_2_ NTs by a dip-coating method. The TiO_2_ NTs were immersed in the drug solution three times, for 5 s each time. The samples comprised three groups: Ti foils (Group 1, control group), Ti foils with no drug-loaded TiO_2_ NTs (Group 2), and Ti foils with PGLa-loaded TiO_2_ NTs (Group 3).

### 2.2 Structural characterization of the three different Ti substrates

The structural characterization of the prepared TiO_2_ NTs, before and after drug loading was performed through field-emission scanning electron microscopy (SEM, S520, HITACHI, Japan). Each Ti sample was fixed with 2.5% glutaraldehyde, treated with osmium acid, dehydrated with serially diluted ethanol, dried in a vacuum, and coated with gold for electron microscope scanning (Nie et al., 2016). The surface morphology of the sample was observed by atomic force microscope (LensAFM Nanosurf, Switzerland). Scanning area was 10 mm by 10 mm. The material roughness was measured and analyzed by the built-in imaging system. To evaulate the wettability of the three Ti substrates, water droplet contact angle were measured with equipment (OCA50, DPIC, Germany) and analysised with a image tool called SCA20 contact angle measurement software. Dosing rate was 1 μl/s, and the water was administered continuously for 10 s.

### 2.3 Biocompatibility evaluation

#### 2.3.1 Cell adhesion and proliferation ability assay

The osteoblast-like MG-63 cells were considered as research models of osteoblasts (Dong et al., 2016). The osteoblast-like MG-63 (osteosarcoma) cell lines were purchased from Cell Bank, Chinese Academy of Science (Shanghai, China). After filtration and antibiotic treatment, the MG-63 cells were cultured in a CO_2_ incubator. After 3 days, the free cells were washed with PBS. The medium was refreshed once in 3 days. The cells were trypsinized with 0.25% trypsin ethylenediaminetetraacetic acid (Solarbio, China), and then the fourth-generation cells were collected for experiments.

The three Ti substrates were collected with 10 ml of cell suspension (1 × 10^5^ cells/ml) respectively into a 50 ml centrifuge tube. The centrifuge tube was placed in an incubator at 37°C and rotated at a uniform speed. Cell counts were measured after 1, 2, and 4 h respectively to measure the adhesion ability of cells on different surfaces. A 100 μl portion of cell suspension (1 × 10^5^ cells/ml) were inoculated onto each Ti substrates and incubated in a 5% CO_2_ incubator for 30 min to allow cells to adhere to the material surface. The samples were added 1 ml of medium and further cultured for 1 day, 3 days, and 5 days. Quantitative analysis in cell adhesion and cell proliferation were measured with a Cell Counting Kit-8 (WST-8/CCK8 ab228554, Abcam, Britain) according to the manufacturer’s instructions.

#### 2.3.2 Cytoskeletal evaluation using phalloidin staining

The effect of each Ti substrate on the cytoskeleton of MG-63 cells was evaluated with phalloidin staining. The cells were seeded according to 1 × 10^4^ cells/well (cell culture plate of 24 wells), then were co-cultured with each group for 24 h (ten pieces in each group). Subsequently, the cells were isolated with PBS and were fixed using 1.2% paraformaldehyde at room temperature for 15 min. The cells were permeabilized with 0.1% Triton X-100 for 1 min at room temperature. After permeabilization, actin filaments of the cells were labeled with Rhodamine fluorescent group and the nuclei were counterstained with DAPI (5 μg/ml). The cells were observed using blue (DAPI) and red filters (Rhodamine) under a fluorescence microscope (OLYMPUS, BX51, Japan).

#### 2.3.3 Alkaline phosphatase activity assay

The alkaline phosphatase test kit (LabAssayTM ALP Kit WAKO, Japan) with P-nitrophenylphosphate as the substrate was used in this study. The osteoblast-like cells were seeded onto each Ti substrates (1 × 10^4^ cells/well). After culturing for 7 days, 20 μl of sample was extracted and added to 96 micorporous plate with 100 μl of substrate buffer. The sample was fully shaken on the plate oscillator for 1 min and incubated at 37°C for 15 min. 80 μl of the reaction termination solution were added, and was fully shaken on a microporous plate oscillator for 1 min. Then the absorbance value at 405 nm was measured with a microplate reader to calculate the alkaline phosphatase activity in the sample.

### 2.4 Antibacterial assay

Three healthy volunteers (without periodontal disease) were selected, and subgingival plaque from the maxillary molars was collected using curettage. This study was approved by the Medical Ethics Committee of Aerospace Center Hospital. The collected plaque was transferred directly to a centrifuge tube containing 5 ml BHI and mixed evenly. The concentration of the solution was determined to be 10^5^ CFU/ml using the McIntosh turbidimetric method in the anaerobic culture at 37°C. Conventional Ti foils, TiO_2_ NTs, and PGLa-loaded TiO_2_ NTs (10 pieces each) were placed in a 24-well plate containing 1.8 ml BHI, and 0.2 ml mixed bacterial solution at a concentration of at least 10^5^ CFU/ml was added. After 24 h of anaerobic culture, the Ti substrates were observed under an electron microscope. In order to further evaluate the antibacterial performance of the Ti substrates, Spread plate counting method was used to evaluate antibacterial abilities of the Ti substrates. The living bacteria in the sample were rinsed, collected and quantified by standard serial dilution and spread plate method. The Ti foil group was used as the control group. The number of viable bacteria in the Ti foil group subtracted the number of viable bacteria in experimental groups (TiO_2_ NTs group and the PGLa-loaded TiO_2_ NTs group) respectively and divided by the number of viable bacteria in the Ti foil group. The results obtained were experimental groups respective antibacterial rates.

### 2.5 Anti-inflammatory assay

#### 2.5.1 Observation of cell morphology in inflammatory environment

The cells were seeded on the plate of 24 wells (1 × 10^4^ cells/well). The inflammatory microenvironment was established by adding TNF-α (10 ng, Novoprotein, Shanghai, China) to the culture medium (maintain a concentration of 0.1 ng/ml TNF-α in the culture environment). The samples comprised three groups: TiO_2_ NTs alone (Group 4, control group), post-treatment with TNF-α (Group 5), post-treatment with TNF-α and PGLa (Group 6). SEM was applied to evaluate the morphology of MG-63 cells on the samples of the three groups (Group 4, Group 5, and Group 6). The MG-63 cells were co-cultured on the samples of the three groups for 24 h and then fixed with 4% paraformaldehyde for 20 min. The samples in the three groups were processed in the manner mentioned above.

#### 2.5.2 Rate of dead cells evaluated with Calcein-AM/PI staining

Calcein-AM/PI staining was performed to evaluate the rate of dead cells per field. The live cells could transfer non-fluorescent Calcein AM to fluorescent green. The living cells emitted green fluorescence and dead cells emitted red fluorescence. The number of red spots was positively correlated with the number of dead cells. The cells were seeded according to 1 × 10^4^ cells/well and were incubated in a 5% CO_2_ incubator at 37°C for 24 h, after which they were co-cultured with samples of the six groups for 24 h. The cells were isolated with trypsin, mixed with PBS containing 1 μg/ml Calcein AM, and then cultured for 30 min at room temperature. The resuspended cells were added to 0.5 μl of PI cells and further incubated for 15 min in the dark. Three visual fields were randomly selected under a fluorescence microscope (magnification, ×100), and the number of living cells and dead cells in the visual field were calculated. The rate of dead cells represented apoptosis, which was calculated using the following equation: rate of dead cells (%) = (number of dead cells/the total cell number) ×100%.

### 2.6 Statistical evaluation

All data were expressed as the mean ± standard deviation and analyzed by one-way ANOVA combined with Student’s *t*-test using SPSS 20.0 (SPSS, United States). A *p*-value less than 0.05 was considered to be statistically significant.

## 3 Results

### 3.1 General observation of each Ti substrate

**FIGURE 1:**
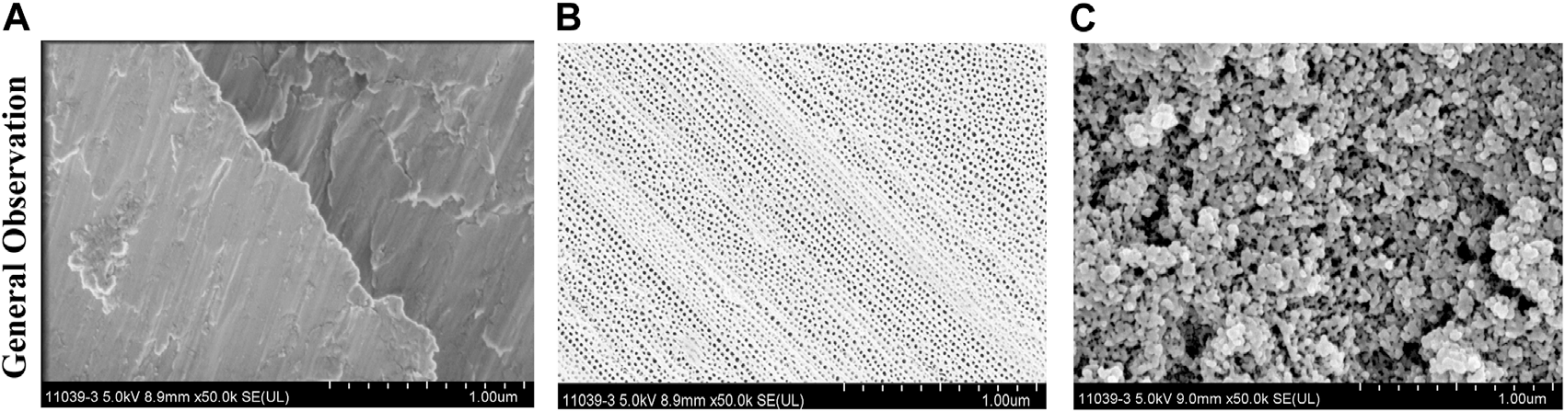
Structural characterizations of the three Ti substrates through SEM: **(A)** Ti foil before anodic oxidation, **(B)** TiO_2_ NTs, and **(C)** TiO_2_ NTs + PGLa. The scale bar represents 1 μm.

The three different Ti substrates were confirmed by SEM, as shown in Figure 1. TiO_2_ NTs were successfully prepared on the surface of the Ti foil by anodic oxidation, and PGLa was loaded into their inner spaces. Figure 1 showed SEM images of the pure Ti foil (a), TiO_2_ NTs (b), and TiO_2_ NTs loaded with PGLa (c). The cavities of the TiO_2_ NTs were evident and open. The structural characteristics of the TiO_2_ NTs were very suitable for drug loading. Figure 1C showed the plane views of the PGLa-loaded TiO_2_ NTs. The cavities of the drug-loaded TiO_2_ NTs were filled with PGLa. Some PGLa clots were observed on the TiO_2_ NTs, as shown in Figure 1C.

**FIGURE 2:**
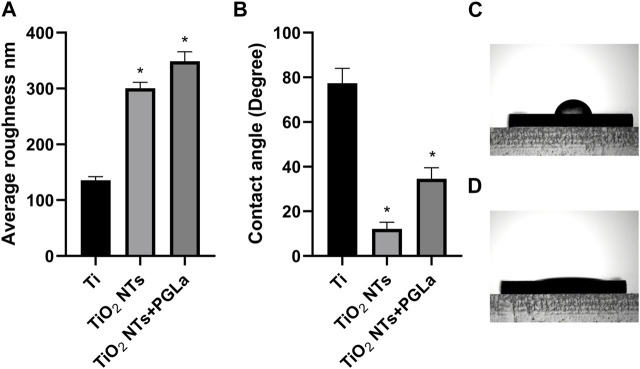
**(A)**: roughness of each Ti substrates: Ti, TiO_2_ NTs, and TiO_2_ NTs + PGLa. **(B)**: contact angle of each Ti substrates: Ti, TiO_2_ NTs, and TiO_2_ NTs + PGLa. The data are presented as the mean ± SD; *n* = 10. *p < 0.05 vs. control (Ti group). **(C)** showed contact angle of Ti foil. **(D)** showed contact angle of TiO_2_ NTs.

Surface roughness and wettability played an important role in osseointegration. The surface roughness and wettability of different materials were displayed through the bar graph in Figures 2A, B. The average surface roughness of untreated Ti, TiO_2_ NTs, PGLa-loaded TiO_2_ NTs were found to be 135.8 ± 6.4 nm, 300.5 ± 10.5 nm, 348.9 ± 16.9 nm, respectively. The contact angle of the untreated Ti was 77.4° ± 6.6° (As shown in the Figures 2B, C). TiO_2_ NTs displayed excellent wettability which of contact angle was 12.1° ± 2.9° (As shown in the Figures 2B, D). The contact angle of the PGLa-loaded TiO_2_ NTs was 34.6° ± 4.9°.

### 3.2 Biocompatibility evaluation

#### 3.2.1 Cell adhesion and proliferation ability assay

**FIGURE 3:**
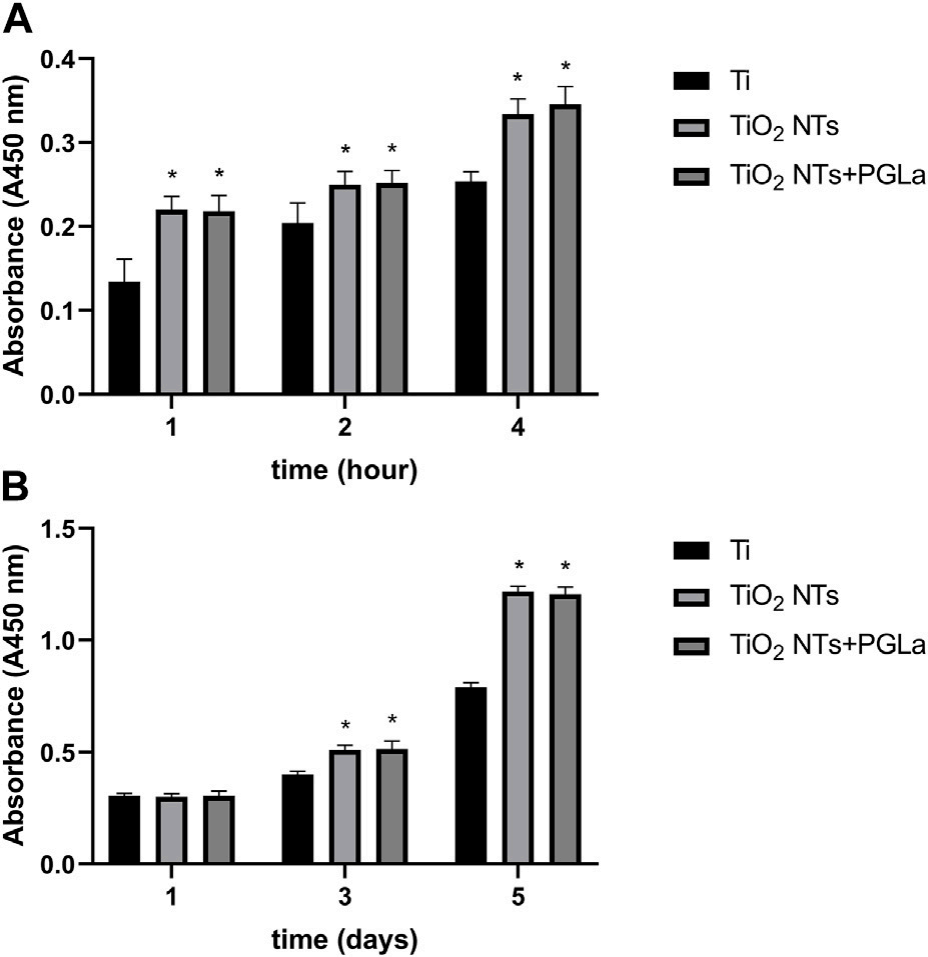
The result of osteoblast-like cells adhesion **(A)** and proliferation **(B)** on each Ti substrates: Ti, TiO_2_ NTs, and TiO_2_ NTs + PGLa. *p < 0.05 vs. control (Ti group).

The histogram in Figure 3A represents the relative amount of cell adhesion of different materials at different time points (1, 2, and 4 h). The number of adherent cells increased with time. Compared with untreated Ti, it was easier for cells to adhere onto the surface of TiO_2_ NTs and PGLa-loaded TiO_2_ NTs. Figure 3B showed the relative amount of cell proliferation of each Ti substrate at different time points (1 day, 3 days, 5 days). The cells on the surface of TiO_2_ NTs and PGLa-loaded TiO_2_ NTs showed stronger proliferation ability.

#### 3.2.2 Cytoskeletal evaluation with phalloidin staining

**FIGURE 4:**
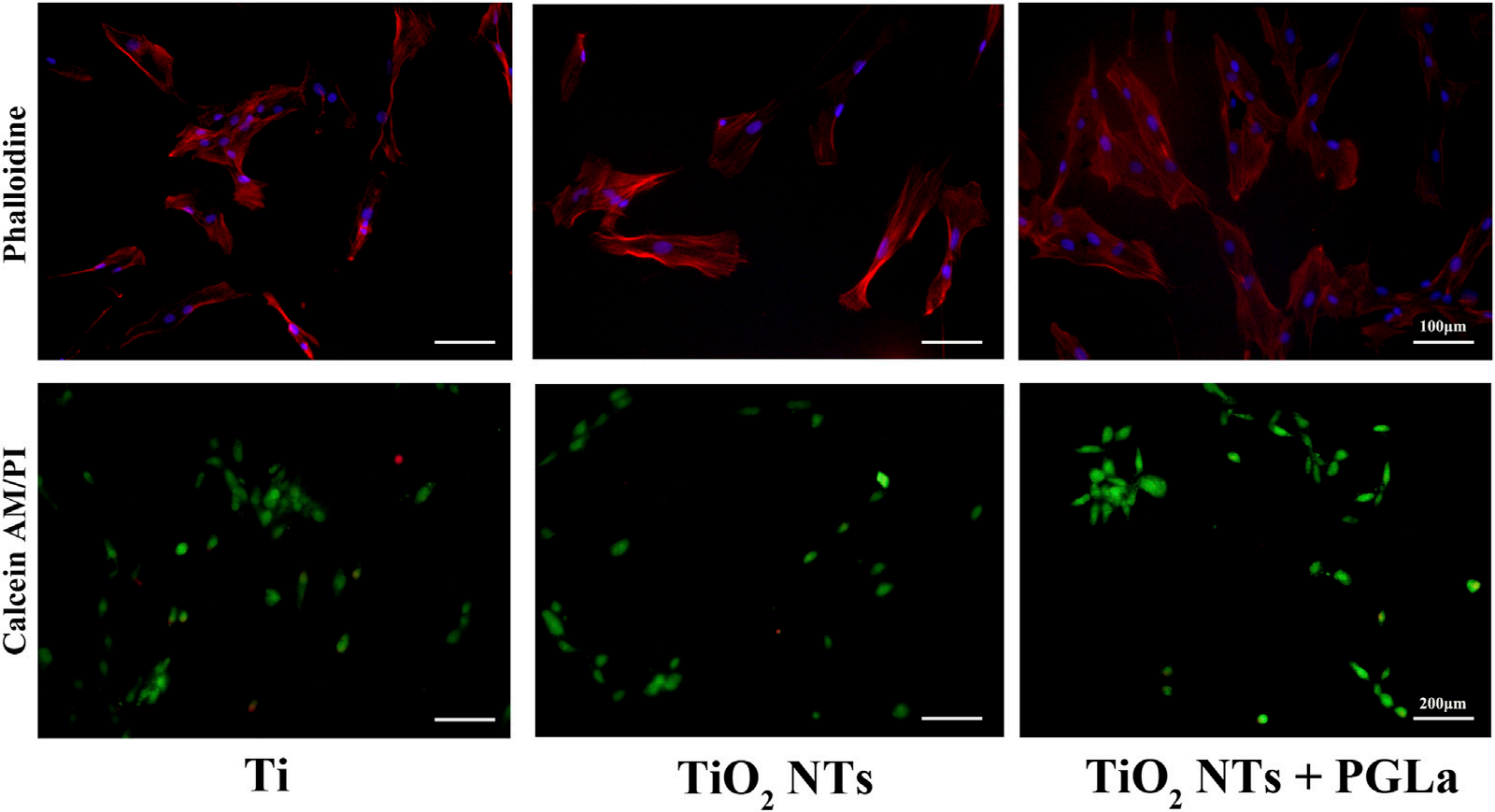
Fluorescent microscopic images of phalloidin staining and Calcein-AM/PI staining for the three Ti substrates: Ti, TiO_2_ NTs, and TiO_2_ NTs + PGLa. Phalloidin staining (magnification, ×200), Calcein-AM/PI staining (magnification, ×100).

Phalloidin staining was used to evaluate whether the cytoskeleton was damaged. In all samples of each group, the results of phalloidin staining showed an intact cytoskeleton. The images in Figure 4 displayed that morphology of actin filaments showed no significant difference among the three groups. DAPI staining showed complete nucleis in all samples of each group.

#### 3.2.3 Alkaline phosphatase activity assay

**FIGURE 5:**
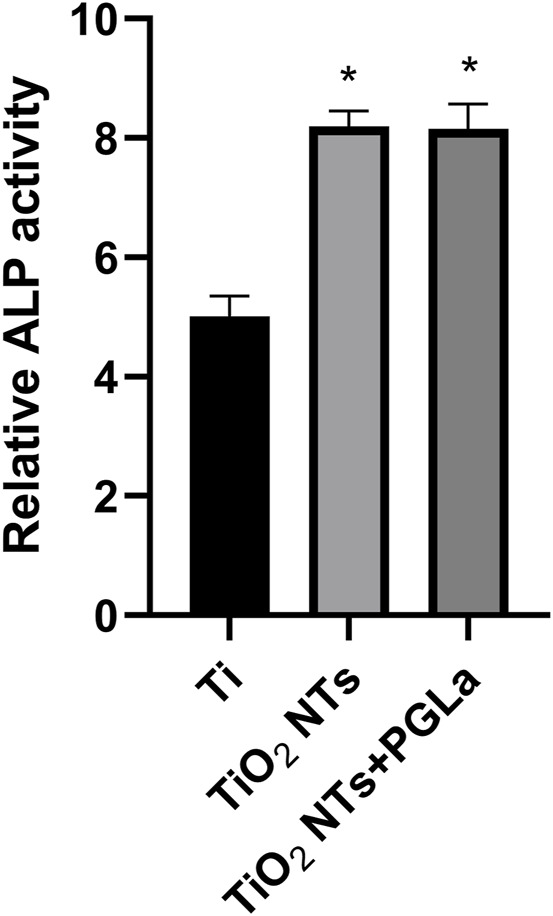
Osteoblast-like cell ALP activity on each Ti substrates after incubation for 7 days. *p < 0.05 vs. control (Ti group).

Figure 5 showed the relative activity of alkaline phosphatase of osteoblast-like cells on the surface of the three Ti substrates. Compared with untreated Ti, the relative activity of alkaline phosphatase of cells on the surface of the TiO_2_ NTs and PGLa-loaded TiO_2_ NTs showed stronger. However, there was no statistically significant difference between the TiO_2_ NTs and PGLa-loaded TiO_2_ NTs.

### 3.3 Antibacterial properties of each Ti substrate

**FIGURE 6:**
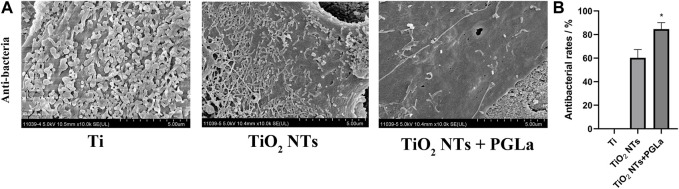
**(A)**: SEM images of bacteria colonization on the surface of each Ti substrate: Ti, entire surface; TiO_2_ NTs, TiO_2_ NT array surface; TiO_2_ NTs + PGLa, TiO_2_ NT arrays loaded with PGLa surface. The scale bar represents 5 μm. **(B)**: Antibacterial rate of TiO_2_ NTs and PGLa loaded TiO_2_ NTs, respectively. *p < 0.05 vs. TiO_2_ NTs group.

The results demonstrate that the samples in the control group were filled with dense bacteria and colonies, and the TiO_2_ NTs surfaces had relatively low levels of adherent bacteria by comparison. Further, the PGLa-loaded TiO_2_ NTs surfaces had the lowest levels of adherent bacteria at 24 h (Figure 6A). The antibacterial rate of TiO_2_ NT group was 60.3% ± 7.0%. The antibacterial rate of PGLa-loaded TiO_2_ NTs group was 84.6% ± 5.5%. The difference between the two groups was statistically significant (*p* < 0.05) (Figure 6B).

### 3.4 Anti-inflammatory assay

#### 3.4.1 Observation of cell morphology in inflammatory environment

**FIGURE 7:**
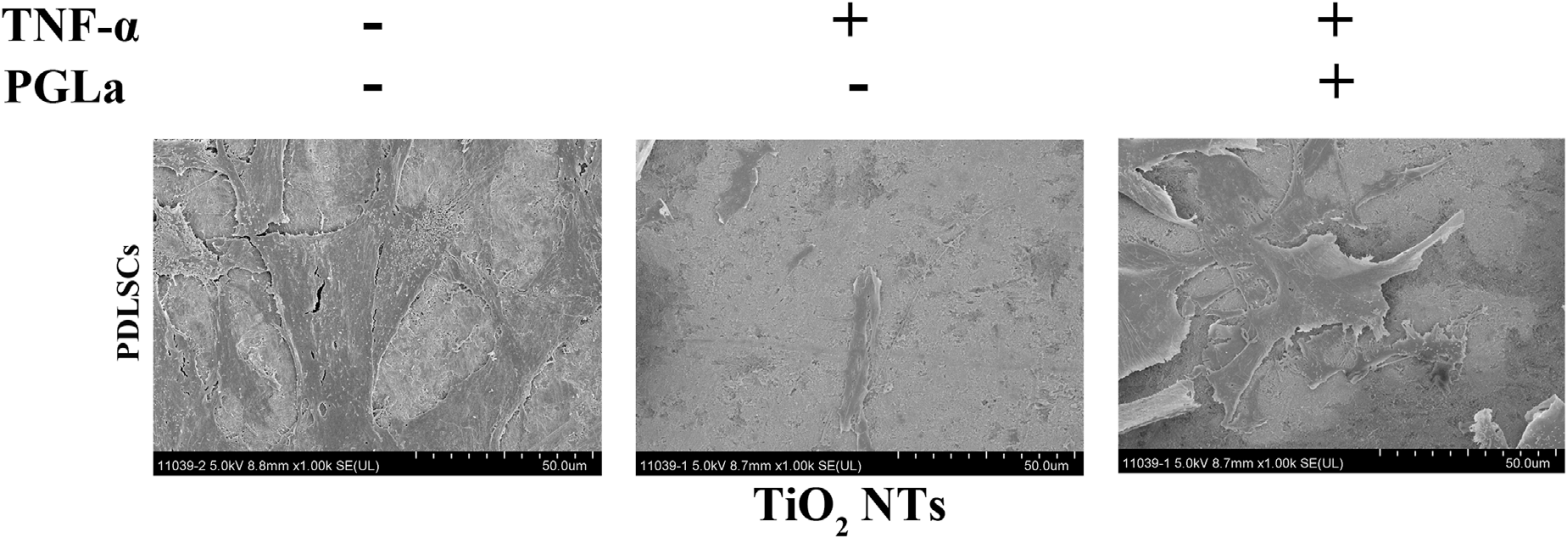
SEM images of the cell morphology of MG-63 cells on the TiO_2_ NTs after 24 h: control (TiO_2_ NTs alone), post-treatment with TNF-α, and post-treatment with TNF-α and PGLa. The scale bar represents 50 μm.

The cell morphology of the MG-63 cells on the substrates was appraised with SEM, as shown in Figure 7. More extensive ranges of filopodia and lamellipodia and more cross-linked cells are observed on the surfaces of the TiO_2_ NTs without TNF-α and PGLa-loaded TiO_2_ NTs with TNF-α compared to the surfaces of the TiO_2_ NTs with TNF-α.

#### 3.4.2 Rate of dead cells evaluated with Calcein-AM/PI staining

**FIGURE 8:**
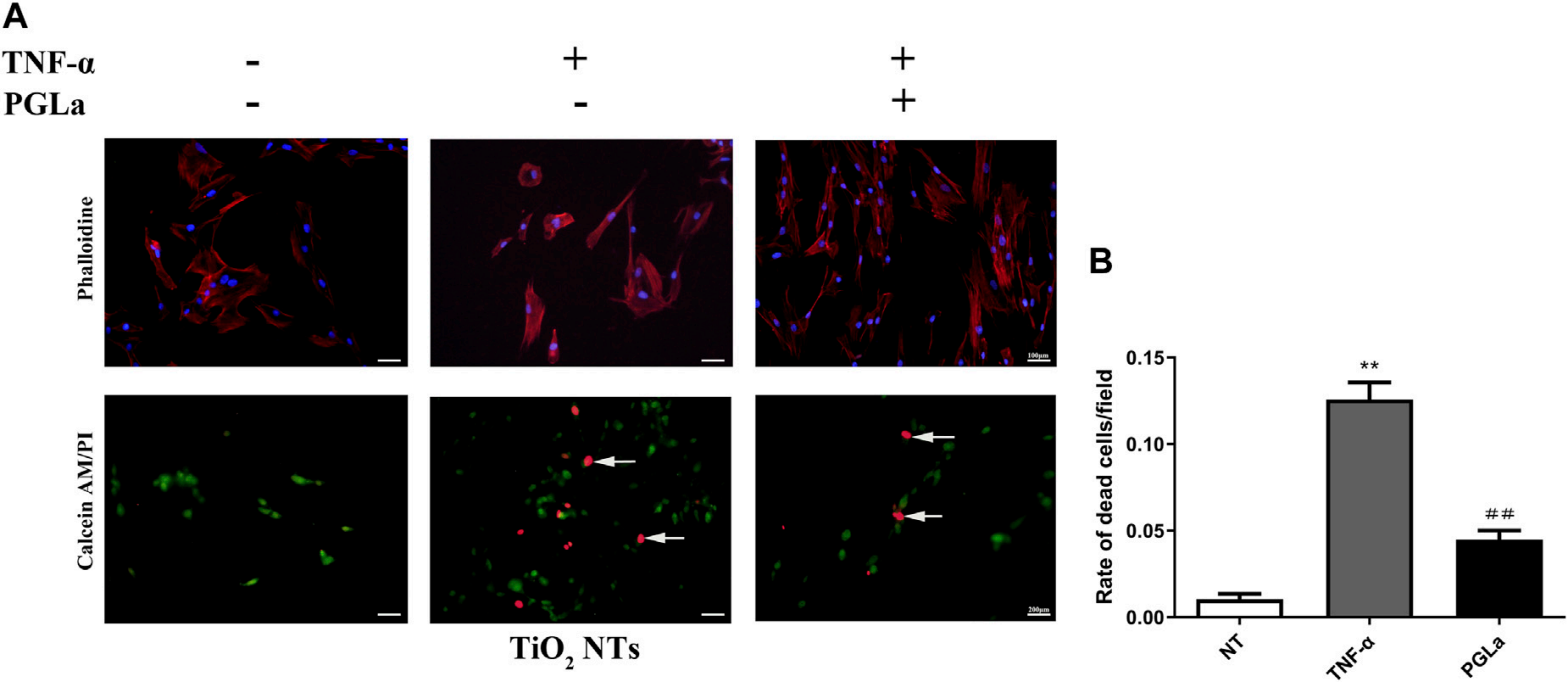
**(A)**: Fluorescent microscopic images of phalloidin staining and Calcein-AM/PI staining for TiO_2_ NTs: control (TiO_2_ NTs alone), post-treatment with TNF-α, and post-treatment with TNF-α and PGLa. Phalloidin staining (magnification, ×200), Calcein-AM/PI staining (magnification, ×100). Arrows indicated dead cells. **(B)**: Calcein AM/PI staining analysis showing the rate of dead cells/field for TiO_2_ NTs: control (TiO_2_ NTs alone), post-treatment with TNF-α, and post-treatment with TNF-α and PGLa. The data are presented as the mean ± SD; *n* = 10. **p < 0.01 vs. control, ^##^ *p* < 0.01 vs. post-treatment with TNF-α.

The dead cells showed fluorescent red under Calcein-AM/PI staining. Without inflammatory factors, the field of vision was full of live cells and few dead cells (Figure 4). On the surfaces of the TiO_2_ NTs without inflammatory factors, the rate of dead cells per field is 1.03% ± 0.01%, and in those with inflammatory factors (TNF-α), the rate of dead cells per field is 12.56% ± 0.03%. The rate of dead cells increased significantly with the addition of TNF-α (*p* < 0.01). On the surfaces of the PGLa-loaded TiO_2_ NTs with inflammatory factors (TNF-α), the rate of dead cells per field is 4.49% ± 0.02%. PGLa significantly reduces the rate of dead cells in the inflammatory microenvironment (*p* < 0.01, Figure 8).

## 4 Discussion

Improving the interaction between the implant and surrounding hard tissue was essential for the long-term success of the implant. In order to reduce the damage of implants from inflammation and microorganisms, TiO_2_ NTs prepared on the surfaces of implants could improve the biological responses of osseointegration, providing a good platform for drug loading (Cha et al., 2016).

TiO_2_ NTs had large specific surface areas and suitable properties as bone contact materials. Anodizing can produce controllable and uniform TiO_2_ NTs of various sizes on the TiO_2_ surface layer. As shown in Figure 1, the TiO_2_ NTs were open at the top (Nemati and Hadjizadeh, 2017). Therefore, they can be loaded with the appropriate drugs for dental application. They were fabricated for use as a drug-loading layer; after drug loading in the TiO_2_ NTs and drying, most were clogged with the drugs, as shown in Figure 1. Drug loading on TiO_2_ NTs can enable sustained release (Kim et al., 2019). Surface roughness and wettability played an important role in osseointegration. The rough surface of dental implants is conducive to the adhesion of osteoblasts, thus facilitating the process of osseointegration. The result of this study showed that more cells adhere to the surface of TiO_2_ NTs and PGLa-loaded TiO_2_ NTs in a short time compared with untreated Ti. However, the surface roughness of TiO_2_ NTs and PGLa-loaded TiO_2_ NTs was not ideal roughness. At present, the surface roughness of mainstream implants was the moderately rough (1∼2 μm) and minimally rough (0.5∼1 μm) (De Bruyn et al., 2000). The roughness of TiO_2_ NTs and PGLa-loaded TiO_2_ NTs was approximately 300∼350 nm. More appropriate preparation parameters need to be further discussed to obtain more ideal roughness. Most studies found that hydrophilic surfaces tend to enhance early cell adhesion, proliferation, differentiation and bone mineralization compared with hydrophobic surfaces (Bornstein et al., 2008). Wettability was directly measured by liquid-solid contact angle, which affected the bioligical response to the implant. Contact angle below 90° indicated that the surface was hydrophilic, and contact angle close to 0° indicated that the surface was superhydrophilic. The results of this study showed that the surface of TiO_2_ NTs and PGLa-loaded TiO_2_ NTs showed better hydrophilicity. Under the influence of better hydrophilicity, the osteoblast-like cells on the surface of TiO_2_ NTs and PGLa-loaded TiO_2_ NTs showed stronger cell proliferation and osteogenic activity. The results revealed that TiO_2_ NTs and PGLa had good biocompatibility.

As an antimicrobial peptide, PGLa was firstly found in the secretion of frog skin (Pachler et al., 2019). The antibacterial properties of PGLa were reflected in their potent inhibition of the activities of *Streptococcus* mutants and *Fusobacterium* nucleate. In addition, by binding with lipopolysaccharide, PGLa showed inhibitory effects on *Escherichia coli*, *Pseudomonas aeruginosa*, and *Porphyromonas gingivalis*. Therefore, PGLa was expected to become a local drug choice for the treatment of bacterial-related oral diseases (McLean et al., 2014).

Subgingival plaque was the most critical factor causing peri-implantitis. Its structure was complex and composed of multiple microbial communities. It could adhere to the surfaces of teeth or implants. Subgingival plaque was difficult to wash away with water (Sbordone and Bortolaia, 2003). Therefore, we used the subgingival plaque of maxillary molars to simulate the *in vitro* environment of peri-implantitis-associated biofilm. In this study, the results revealed relatively few bacteria on the TiO_2_ NTs. Previous studies have shown that TiO_2_ NTs could inhibit bacterial colonization and reduce the number of microorganisms on their surfaces (Peng et al., 2013). The PGLa-loaded TiO_2_ NTs surfaces had a minimum number of adherent bacteria at 24 h of co-culture, as PGLa surfaces can enhance antibacterial ability.

Pathogenic bacteria caused immune and inflammatory reactions in the tissue around implants. The immune system of the body resisted the bacteria and isolated the diseased tissue, eventually leading to peri-implantitis. Inflammatory factors around implants can induce monocytes to differentiate into osteoclasts, resulting in bone destruction around implants. There was a lack of tissue similar to the periodontal ligament that could provide stem cells or blood supply, leading to extensive and rapid bone loss in peri-implantitis (Park et al., 2020; Nagatomo et al., 2006). Most scholars had found that osteoblast played an essential role in alveolar bone regeneration and repair (Choi et al., 2013).

Actin filaments can arrange cellular contents and change shapes, forming the structural framework of cells. The cytoskeletal integrity played a significant role in mechanical properties and cell functions (Svitkina, 2018). The results of phalloidin staining of MG-63 cells treated with each Ti substrate showed that the morphology of actin filaments was normal. The DAPI staining of MG-63 cells treated with each Ti substrate showed intact nucleus. The results also revealed that TiO_2_ NTs and PGLa had good biocompatibility and did not cause any damage to the cell viability. On the surface of each Ti substrate without inflammatory factors, the field of vision was full of live cells and few dead cells. These results represent a promising step toward the application of TiO_2_ NTs and PGLa without eliciting any toxic response.

As an inflammatory cytokine, TNF-α played an essential role in the bone destruction of peri-implant inflammation (Petkovic-Curcin et al., 2017; Duarte et al., 2009). The concentration of TNF-α in the gingival crevicular fluid determined the degree of periodontal inflammation (Liu et al., 2018). TNF-α regulated cell proliferation, differentiation, and apoptosis by binding to membrane-bound receptors (Locksley et al., 2001). The addition of TNF-α to the culture medium of MG-63 cells simulated the inflammatory microenvironment around the implant. The rate of dead cells/fields increased significantly on the surface of the TiO_2_ NTs with TNF-α. The increase in TNF-α levels leaded to increased apoptosis in MG-63 cells. To some extent, it simulated the development of peri-implant inflammation. However, with the addition of PGLa, the rate of dead cells/fields was significantly reduced, which indicated that PGLa had an anti-inflammatory effect and can alleviate the inflammatory state of MG-63 cells.

In this study, the biocompatibility, antibacterial and anti-inflammatory effects of PGLa-loaded TiO_2_ NTs were preliminarily evaluated. However, the optimal dosage of PGLa and its local pharmacokinetics should be further studied.

## 5 Conclusion

We prepared TiO_2_ NTs on Ti substrates by electrode oxidation. The TiO_2_ NTs were loaded with PGLa for drug delivery. Then, we observed the microstructures of different Ti substrates and studied the antibacterial and anti-inflammatory effects of these substrates. The SEM results showed the surface morphology of TiO_2_ NTs and the successful loading of PGLa drug layers. PGLa-loaded TiO_2_ NTs showed good biocompatibility. The PGLa-loaded TiO_2_ NTs significantly reduced the number of bacterial colonization on the surface of the Ti substrate. Furthermore, this improved Ti substrate could alleviate the inflammatory response induced by TNF-α, reducing the damage of inflammation on MG-63 cells significantly. Therefore, it could be concluded that the PGLa-loaded TiO_2_ NTs had excellent application prospects in dental implants.

## Data Availability

The original contributions presented in the study are included in the article/supplementary material, further inquiries can be directed to the corresponding author.
